# The TALE Transcription Factor Homothorax Functions to Assemble Heterochromatin during *Drosophila* Embryogenesis

**DOI:** 10.1371/journal.pone.0120662

**Published:** 2015-03-20

**Authors:** Miguel Angel Zaballos, Walter Cantero, Natalia Azpiazu

**Affiliations:** Centro de Biología Molecular “Severo Ochoa” CSIC-UAM, C/ Nicolás Cabrera, 1 Universidad Autónoma de Madrid, Madrid, Spain; University of Florida, UNITED STATES

## Abstract

We have previously identified Homothorax (Hth) as an important factor for the correct assembly of the pericentromeric heterochromatin during the first fast syncytial divisions of the *Drosophila* embryo. Here we have extended our studies to later stages of embryonic development. We were able to show that *hth* mutants exhibit a drastic overall reduction in the tri-methylation of H3 in Lys9, with no reduction of the previous di-methylation. One phenotypic outcome of such a reduction is a genome instability visualized by the many DNA breaks observed in the mutant nuclei. Moreover, loss of Hth leads to the opening of closed heterochromatic regions, including the rDNA genomic region. Our data show that the satellite repeats get transcribed in wild type embryos and that this transcription depends on the presence of Hth, which binds to them as well as to the rDNA region. This work indicates that there is an important role of transcription of non-coding RNAs for constitutive heterochromatin assembly in the *Drosophila* embryo, and suggests that Hth plays an important role in this process.

## Introduction

The eukaryotic genomic DNA is packed into two types of chromatin: the euchromatin and the heterochromatin. Euchromatin is the open state, less condensed and more accessible for regulatory factors that facilitate its transcription. Heterochromatin, on the contrary, is highly condensed and less accessible for transcription. The different packaging of the genomic DNA in *Drosophila* depends basically on histone-modifying enzymes and chromatin-remodelling complexes. Heterochromatin is rich in tandemly repeated sequences and transposable elements, it is characterized by histone methylation and hypoacetylation, and is usually associated with HP1 (heterochromatin protein 1) [1, 2]. Two types of heterochromatin can be found in the cells: facultative and constitutive heterochromatin. The facultative heterochromatin is associated with gene regulation and designates the genomic regions that can adopt open or close conformations depending on temporal and spatial contexts. In contrast to this, constitutive heterochromatin is stable and conserves its heterochromatic conformation during all stages of development and in all tissues. In the past years several works mainly done in yeast have suggested that constitutive heterochromatin establishment requires non-coding RNA transcription [3, 4]. In *Drosophila* a link between non-coding satellite RNA transcription and pericentromeric heterochromatin assembly has also been established [5]. However, very little is known about the regulation of this non-coding RNA transcription and its phenotypic outcome in a developing organism. Work done in mouse cells and in *Drosophila* clearly shows that specific transcription factors are involved in the formation of heterochromatin [5–7].

Homothorax belongs to the TALE-homeodomain subfamily of transcription factors and shares a high degree of homology with their vertebrate counterparts: the Meis family of proto-oncogenes [8–10]. All members of this family have a conserved domain in its N-terminal part, called the HM domain (Homothorax-Meis domain). This domain has been described to be fundamental for the interaction and nuclear translocation of Extradenticle (Exd), another TALE-homeodomain subfamily, which is homologous to Pbx in vertebrates [10–13].

Many diverse functions have been described for these TALE-homeodomain transcription factors during embryonic and adult development, in vertebrates as well as in *Drosophila*, suggesting that they are key developmental regulators.

One of the principal roles described for *hth/exd* is its function as cofactors of Hox proteins [10, 14–17]. In addition to their role as Hox cofactors, there are *hth/exd* functions that seem to be independent of Hox activity. Indeed, during larval development *hth* is involved in the subdivision of wings and legs into proximal and distal domains [18–22], in the development of the posterior part of the notum or *scutellum* [23], and as selector gene in antennal development [24, 25]. Moreover, Hth plays multiple roles in the formation of adult fly organs, both as a selector of identity and an organizer of proximal-distal axis [18, 21, 24, 26] and has been also reported to play a role in cell proliferation [27]. That *hth/exd* have all these functions is not totally unexpected since *hth* and *exd* encode transcription factors that regulate the transcriptional activity of specific targets genes. However, an unexpected role for *hth* in centric heterochromatin assembly in early *Drosophila* embryos has been described [5]. In this context, Hth facilitates the transcription of satellite non-coding RNAs in the pericentromeric region of chromosome X.

In this work we further analyse the role of the transcription factor Hth in constitutive heterochromatin formation during *Drosophila* embryonic development. Our results point to a crucial role of the transcription factor in the correct tri-methylation of the histone H3 in the Lys9 residue, a histone mark that is associated with highly compacted genomic regions in *Drosophila*.

## Materials and Methods

### Drosophila strains

The following *Drosophila* strains were used in this work to analyse mutant phenotypes or generate loss of function clones:

Ovoflp; FRT82BDfhth/TM6 and FRT82BovoD1/ßtub85De/TM3,Sb (Bloomington). We used the Df(3R)hth from Exelisis (6158), which eliminates almost all hth transcripts, to generate the FRT82hthDfhth/TM6 recombinant, and the *hth* ^*P2*^ mutant [9].

### Generation of female germ line clones

To generate *hth* female germ line clones ovoflp;FRT82BDfhth/TM6 females were crossed with FRT82BovoD1/TM3 males. Virgin females of the genotype ovoflp;FRT82BDfhth/FRT82BovoD1 were collected and crossed with Dfhth/TM3,GFP and hth^P2^/TM3,GFP males respectively. The embryos laid by the females were collected and fixed for antibody staining.

### Immunostaining of embryos

Embryos were collected, de-chorionated and immediately fixed in a mixture of 5% formaldehyde and heptane for 20 minutes at room temperature. The aqueous phase was removed and methanol added. The vitelline membrane was removed by vigorous shaking and the embryos were washed in methanol several times. They were subsequently rehydrated and blocked in 10% BSA. The incubation with primary antibody was done overnight in PBT (PBS, 0.1% Tween). The antibodies used were: anti-Hth 1:500 (rabbit), anti-H3K9me3 (Millipore) 1:400 (rabbit), anti-H3K9me2 (Cell Signalling) 1:400 (rabbit), anti-Fibrillarin (Novus Biologicals) 1:50 (mouse), anti Histone H2AvD pS137 (Rockland) 1:500 (rabbit). Washes were performed in PBT, and the appropriate fluorescent secondary antibody was added for 1 hour at room temperature. Following further washes in PBT, topro3 was added for 15 mins., washed again, and the embryos were mounted in Vectashield (Vector Laboratories). Images were taken in a confocal laser MicroRadiance microscope (Leica) and subsequently processed using Adobe Photoshop.

### Real-time RT-PCR

RNA was extracted from syncytial embryos using the GE Healthcare extraction kit. The real-time PCR was performed in the presence of SYBR Green on a Roche LightCycler 480 with the following pair of primers:

260*fw*: 5’-TGGAAATTTAATTACGAGCT-3’

260*rv*: 5’-ATGAAACTGTGTTCAACAAT-3’

359*fw*: 5’-GTTTTGAGCAGCTAATTACC-3’

359*rv*: 5’-TATTCTTACATCTATGTGACC-3’

361*fw*: 5’-TGAGCTCGTAATAAAATTTCC-3’

361*rv*: 5’-TCAACGATGTATGACATTCC-3’

To normalize, we used primers for *bicoid*:

bcd*fw*: 5'-AAG GGT CTG GAC AAG AGC TG-3'

bcd*rv*: 5'-AAG GCT CTT ATT CCG GTG CT-3'.

### Chromatin immunoprecipitation (ChIP)

Chromatin immunoprecipitation assays were carried out with DNA obtained from 0.52 g of D. melanogaster 2 hours staged embryos as previously described [28] with some minor modifications. Homogenized embryos were sonicated 5 times [10 seconds continuous pulses at 7 amplitude microns power) in a MSE Soniprep 150 Leubasonifier with a microtip probe at 4°C, with 30 s cooling on ice between pulses, yielding DNA fragments mostly between 200 and 700 bp. For immunoprecipitation, rabbit anti-Homothorax (1:25), rabbit anti-H3K9me3 (1:25) were used. Controls were performed with rabbit pre-immune serum. Immunoprecipitated DNA was used for Real-Time PCR amplification with primers for the different subfamilies of the 1688 satellite region (the same primers described above), and the rDNA region (see below) using a Roche Light Cycler equipment and accessories. The data are presented as the amount of DNA enrichment normalized with the input (100% value).

### DNase I sensitivity assay

DNase I sensitivity assays were performed as previously described [29] with minor modifications. Samples (2 ml each) were analysed by qPCR using Roche Light Cycler equipment. The cycle threshold (CT) value for each locus was obtained using Roche Molecular Biochemical-Light Cycler Software (version 3.5). The euchromatin actin (act5c) locus was amplified using the primers

Ac5Cfw: 5′-CAC GGT ATC GTG ACC AAC TG-3′

Ac5Crv: 5′-GCC ATC TCC TGC TCA AAG TC-3′

The heterochromatin locus was amplified using the primers

H23fw: 5′-CCA AGT TGG CCA GTT TTG AT-3′

H23rv: 5′-AGT TCA AGC CCG GGT ATT CT-3′

1360*fw*: 5’-TGTATCGTTTTTAAAAAATTGTC-3’

1360*rv*: 5’-GTGGACCTGTAATATATGCTCT-3’

The primers used for the satellite regions are the same as in the real time RT-PCR chapter, and the rDNA genomic region was amplified with following primers:

rDNA*fw*: 5’-GGC TAA AAC CAA GCG ATC GC-3’

rDNA*rv*: 5’-TTT TCG TCA CTA CCT CCC CG-3

### Western blot analysis

To perform the western blot analysis differential extraction of embryonic proteins was performed. For the analysis of nuclear and extra-nuclear pools of proteins, embryos were disaggregated in hypotonic lysis buffer (10mM Tris-HCl, pH 7.4, 10mM NaCl, 3mM MgCl_2_, 0,3% (v/v) Nonidet P-40, 2mM Na_3_VO_4_, 10mM NaF and protease inhibitors). They were incubated on ice for 10 mins. to allow lysis, and were then centrifugated at 500xg for 5 mins. to obtain the nuclear fraction. The supernatant (containing a mixture of plasma membrane microsomal vesicles, cytoskeleton, and cytosol) is kept apart. Pellets containing cell nuclei were washed in lysis buffer without Nonidet P-40 and again pelleted at 500xg. Both the extra-nuclear and nuclear fractions were solubilized in 2X Laemmli sample buffer.

To isolate chromatin-bound histones the extraction was performed as described in [30, 31]. The embryos were de-chorionated, homogenized and re-suspended in 1ml of cold lysis buffer (10mM Tris-Cl pH 8.0; 1mM KCl; 1.5mM MgCl_2_; 1 mM DTT; protease inhibitors). They were incubated in the lysis buffer for 30 mins at 4°C. They were subsequently centrifuged (10.000g, 10 min.) and re-suspended in 400μl 0.2N HCl. Incubation with HCL was done o/n at 4°C. Following a 10 min. centrifugation (16.000g) at 4°C the histones were precipitated using 33% TCA and incubating 30 mins. on ice. After 10 min. centrifugation at 4°C (16.000g) the pellet was washed with cold acetone and re-suspended in water (Mili-Q).

All the different protein lysates were run in an acrylamide gel and transferred to nitrocellulose membranes. Those were counter-stained with Ponceau Red to visualize total protein transferred. The antibodies used were: anti-H3K9me2 (Cell signalling), anti-H3K9me3, anti-H3 (Millipore) and anti-tubulin (Sera-Lab). Secondary antibodies were IRDye 800 anti-mouse, IRDye 800 anti-rat and IRDye 680 anti-rabbit from Li-Cor. Membranes were scanned with an Odyssey Infrared Imager.

### In situ hybridization

To generate a DNA probe that hybridizes to the rDNA genomic region a PCR was performed using the same primers as above. The PCR product was purified and DIG-marked using the DIG-labelling kit (Roche).

Embryos were fixed, and stained with the anti-Fibrillarin antibody as explained previously. After the staining, the embryos were incubated for 2mins at 91°C together with the DNA probe as described in [32]. They were incubated o/n at 48°C with the probe. They were then washed 3X30 mins. with hybridization solution (50% formamide, 5X SSC, 50 mg/ml heparin, 0.1% Tween20 and 40 mg/ml tRNA) at 48°C and 4X20 min. with PBT at RT. A 1hr. incubation with an anti-DIG antibody (made in goat) followed by 3X10 min. washes in PBT were performed. The embryos were then incubated 1 hour with a biotinylated anti-goat antibody, washed 3X10mins in PBT and incubated with the streptavidin AB Complex (Vectastain, Vector Laboratories). Following 3X10 min. washes in PBT the embryos were incubated with a FITC-tyramide complex (TSA fluorescein system) for 10 min. further washed in PBT and mount in Vectashield (Vector laboratories).

## Results

### Distribution of the H3K9me2 and H3K9me3 histone marks in *homothorax* mutant embryos

**Fig 1:**
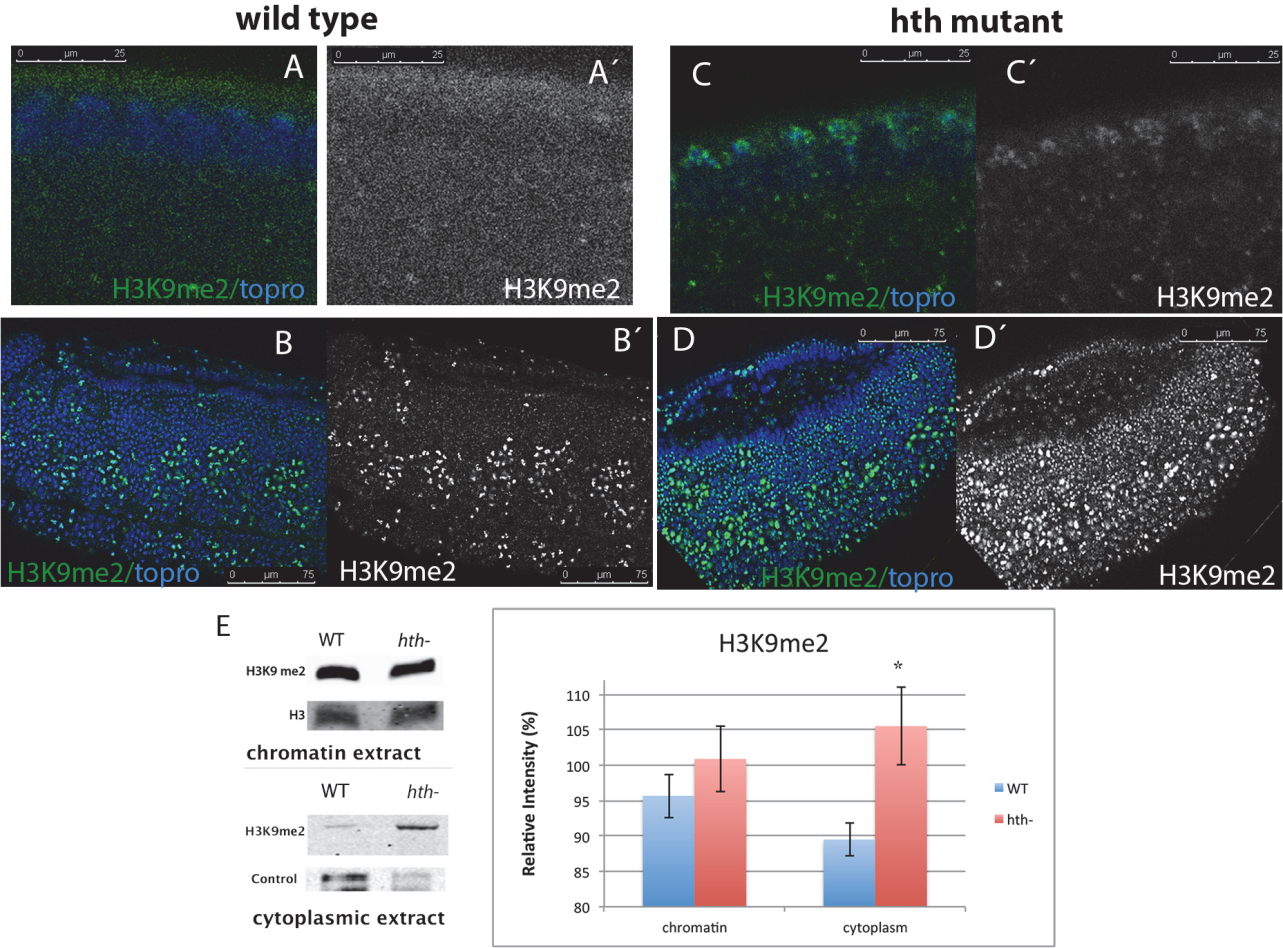
The H3K9me2 methyl mark accumulates in *Dfhth* mutant embryos. A-B) Wild type embryo stained with a specific anti-H3K9me2 antibody (green/grey). A, A´) In a cycle 12 wild type embryo the H3K9me2 mark is not detectable. B, B) In a germ band extended embryo (stg. 11) the H3K9me2 mark is present at high levels in the mitotic nuclei. C-D) *Dfhth* mutant embryo stained as in A. C, C´) Accumulation of H3K9me2 starts to be observed as early as in cycle 11 mutant embryos. D, D´) The H3K9me2 methyl mark is detected at high levels throughout a germ band extended mutant embryo. The mark accumulates in all the nuclei, not only in the mitotic ones. In all figures, embryos are oriented with the anterior to the left and dorsal up. (Embryos were staged as in [61]). E) Western blot analysis of chromatin and cytoplasmic embryonic extracts shows that *Dfhth* embryos have increased levels of H3K9me2 in the cytoplasm, whereas the chromatin bound H3K9me2 does not change with respect to wild type embryos. The western blot was performed three times and with three different sets of extracts to quantify the signal. Quantification is shown in the graph (p-value: 0,02454258 for the cytoplasmic extract and p-value: 0,09575181 for the chromatin extract. They were calculated using the T-Test). Error bars in the graph show the standard deviation. Controls are anti-H3 for the chromatin extract and anti-α-Tubulin for the cytoplasmic extract. Band quantification was done using ImageJ.

In previous work we detected an aberrant distribution of the centromere identifier CID protein in embryos derived from mothers with *homothorax* (*hth*) germ line clones. Our work suggested that *hth* plays an important role in pericentromeric heterochromatin assembly [5]. To investigate if the role of *hth* in heterochromatin formation is more general, we checked the general distribution of heterochromatic marks in Df*hth* embryos derived form mothers with germ line clones (see material and methods). Two specific marks were analysed; the di-methylation of the histone H3 in Lys9 and the tri-methylation of the same histone residue. A previous report has shown that H3K9me2 precedes H3K9me3 in heterochromatin formation during *Drosophila* embryogenesis [33]. It has been described that after removal of the methylation in the Lys4 residue of H3, early *Drosophila* embryos are ready for the subsequent methylation of the same histone in the residue Lys9. This process seems to occur in a sequential manner and the di-methylation of the histone mark is observed slightly before the tri-methylation [33]. To check if *Dfhth* mutant embryos display alterations of this pathway, we decided to analyse the distribution of H3K9me2 in these and compared them with wild type controls. As shown in Fig. 1 the mutants seem to display a higher accumulation of the H3K9me2 mark when compared with wild type embryos (compare Fig. 1A with C and B with D). This accumulation is first detected in the cycle 12 of early syncytial development (Fig. 1 C, C´). In the same developmental stage the signal is not detectable in wild type embryos (Fig. 1 A, A´). Moreover, we were able to visualize high levels of H3K9me2 in the mutant embryos throughout embryonic development (Fig. 1 D, D, compare with 1B,B´). The same observation was made when *hth* ^*P2*^ mutants were stained with the anti-H3K9me2 antibody (S1 Fig.).

To confirm this observation, we performed western blot analysis of chromatin extracted from wild type and *hth* mutant embryos. The membrane was hybridized with a specific antibody that recognizes the di-methyl state of the histone H3 in Lys9. As shown in Fig. 1 E the observed higher accumulation of H3K9me2 in mutant versus wild embryos was not confirmed in the blots performed with the chromatin extract. We suspected that this could be due to a higher accumulation of the di-methyl mark in the cell but not directly associated with chromatin. To analyse if a fraction of H3K9me2 accumulates in the cytosol we performed subcellular fractionation of wild type embryonic extracts to separate cytosolic from nuclear proteins. Western blot analysis of the fractionation using the anti-H3K9me2 antibody shows two different bands of distinct molecular weight. The nuclear fraction displays the expected 17kDa band. In the cytosolic fraction the only band present has a molecular weight of ca. 150kDa (S2 Fig.). We then looked for the presence of both bands in mutant embryos and compare them with their wild type siblings. By doing so we detected a more intense band in the cytosolic fraction of extracts from mutant embryos compared with the wild type ones (see Fig. 1 E cytoplasmic extract). Quantification of the band confirmed that *hth* mutants accumulate more H3K9me2 in the cytoplasm (see Fig. 1).

**Fig 2:**
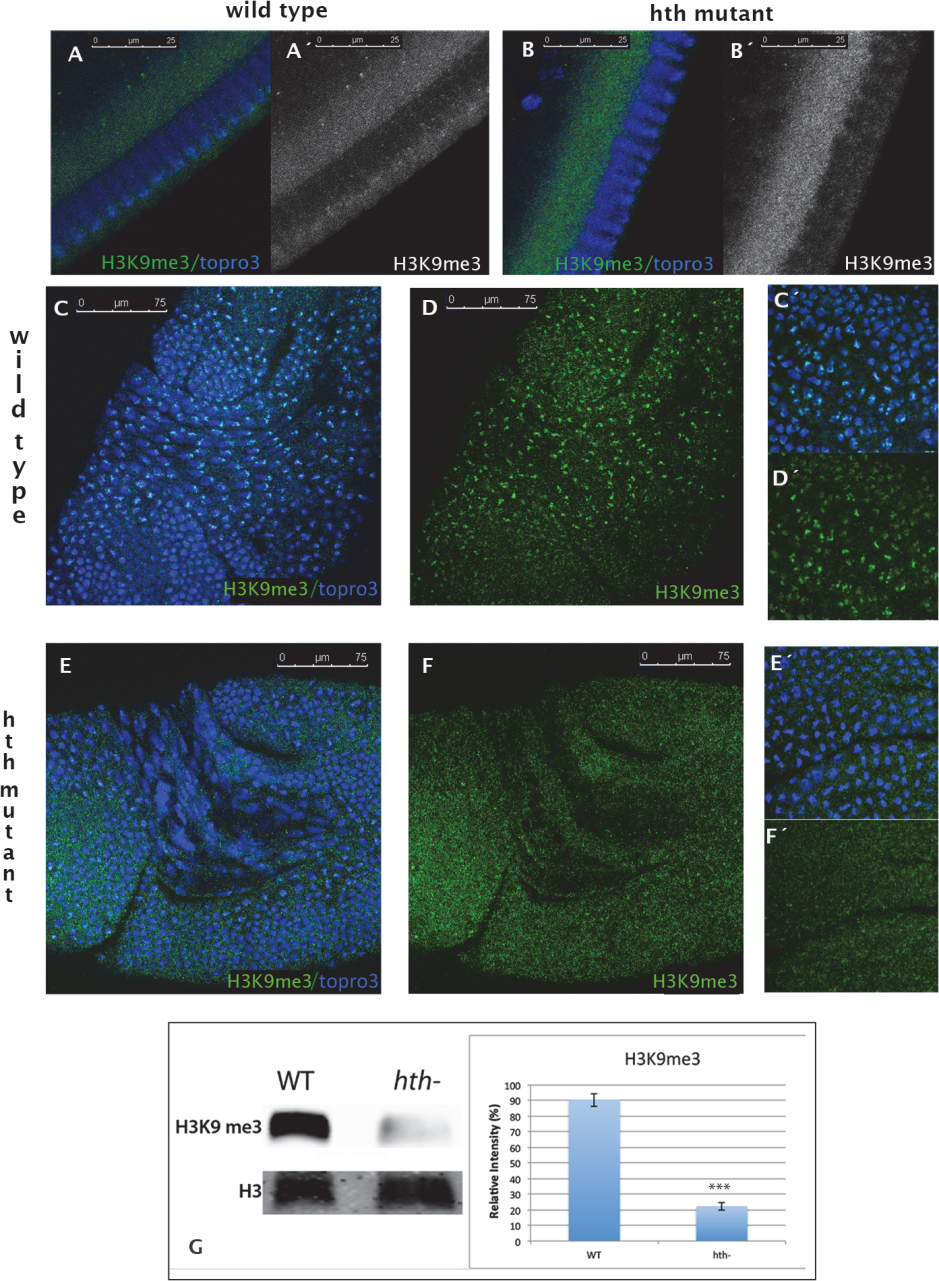
Tri-methylation of histone H3 in Lys9 is severely impaired in *hth* mutant embryos. A, A´) Cycle 14 wild type embryo stained with a specific antibody that recognizes the H3K9me3 mark (green/grey). The signal is detected in the apical domain of the nuclei, which corresponds to the topro3 dense domain. B,B´) Cycle 14 *hth* mutant embryo stained as in A. Note the lack of signal in the nuclei. C, D) Stage 8 embryo stained as in A. High levels of H3K9me3 are observed in all the mitotic cells and lower levels are detected in the heterochromatin domains of the interphase nuclei (see inset C´, D´). E, F) In a stage 8 mutant embryo the levels of H3K9me3 are very much reduced when compared to wild type embryos (see inset F´). (Embryos were staged as in [61]). G) Western blot analysis of chromatin extracts confirms that the chromatin extracted from *hth* mutants show reduced levels of the H3K9me3 methyl mark. The graph represents the quantification of the signal observed. The western was performed three times with three different extractions (p-value: 0,00025212, calculated using T-Test). Error bars in the graph show the standard deviation and have being calculated as in Fig. 1 E. Anti-H3 was used as a loading control. Band quantification was done using ImageJ.

We then looked at the tri-methylation of the same histone residue. In wild type embryos, the first accumulation of H3K9me3 is visible in nuclei of early cycle 14 [33] (see Fig. 2 A, A´). This accumulation fails to occur in *Dfhth* embryos at that same stage of embryonic development (Fig. 2 B, B¨) and *hth* mutants show a drastic reduction of H3K9me3 throughout embryonic development (Fig. 2 E, F compare with C, D). The same reduction was observed in the *hth* ^*P2*^ mutant embryos (S3 Fig.). Western blot analysis of chromatin extracts and band quantifications confirm this result (Fig. 2 G).

### *hth* mutants show increased proportion of DNA breaks

**Fig 3:**
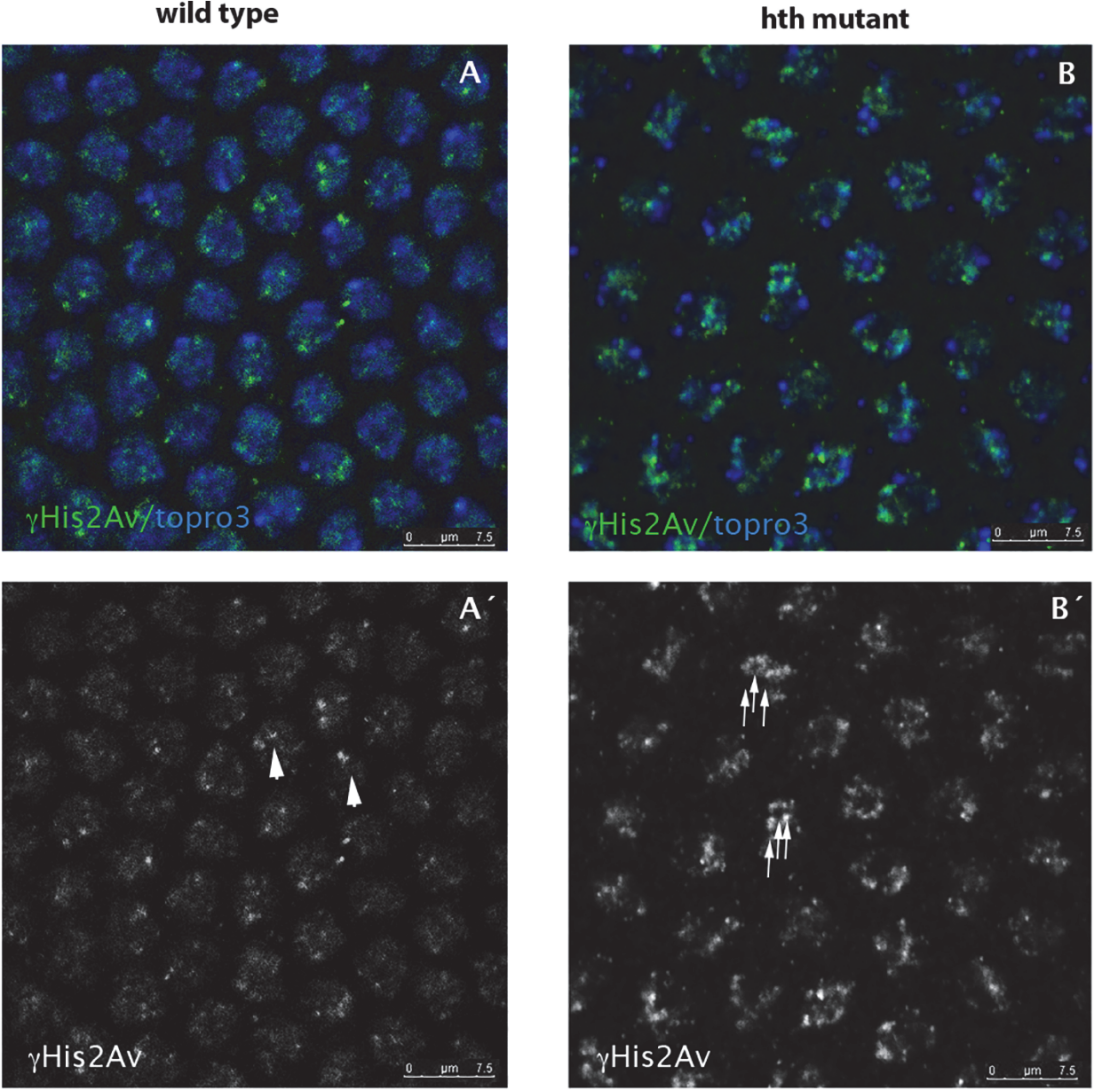
*hth* mutant nuclei show increased number of DNA breaks. A,A´) Wild type syncytium (cycle 11) stained for His2AvP (green) and topro3 (blue). Due to the lack of mitotic checkpoints during the rapid syncytial divisions the nuclei show some degree of DNA breaks marked with the anti-His2AvP antibody (arrowheads in A´). B, B´) However, *hth* mutant nuclei show more nuclear signal when stained with the same antibody (see arrows in B´) indicating that they have more breaks in their DNA. C) Quantification of the DNA breaks in the Dfhth mutant embryos and in their wild type siblings. Quantification was performed by counting the number of nuclear dots marked with the anti-His2AvP antibody. (N = 50 nuclei for each genotype from 5 different embryos each. P-value: 7,0085X10^−5^).

It has been previously reported that mutants for the methyl-transferase Su(var)3–9 show genome instability [34, 35]. These embryos, with reduced levels of H3K9me2, display increased spontaneous DNA-damage. In our mutants, the di-methylation of H3 in K9 is not reduced, but the levels of tri-methylation are. To address if the diminution in the levels of H3K9me3 has an effect on genome stability, we examined the degree of spontaneous DNA damage that occurs in Df*hth* mutant embryos using an antibody specific for γH2Av (the phosphorylated form of the histone variant H2Av at serine 137), which has been shown to associate with DNA repair sites [36]. Early syncytial embryos do not have checkpoints during their fast mitotic divisions, and as a consequence, the number of DNA breaks is higher than in cellularized embryos. We observed that *hth* mutants have increased frequencies of γH2Av marks in the nuclei as compared with their wild type siblings (see Fig. 3, arrows in B´ and compare with arrowheads in A´), indicating that the mutant embryos display a higher frequency of spontaneous DNA breaks. The result was quantified counting the number of dots marked with anti-His2AvP in wild type and in mutant nuclei (Fig. 3 C). We detected the same higher frequency of γH2Av marks in *hth* ^*P2*^ mutants (S4 Fig.).

This result also suggests that the sole presence of H3K9me2 in the chromatin is not sufficient to avoid genome instability during the early embryonic development.

### Heterochromatin in the rDNA is tri-methylated in Lys 9 of histone 3

**Fig 4:**
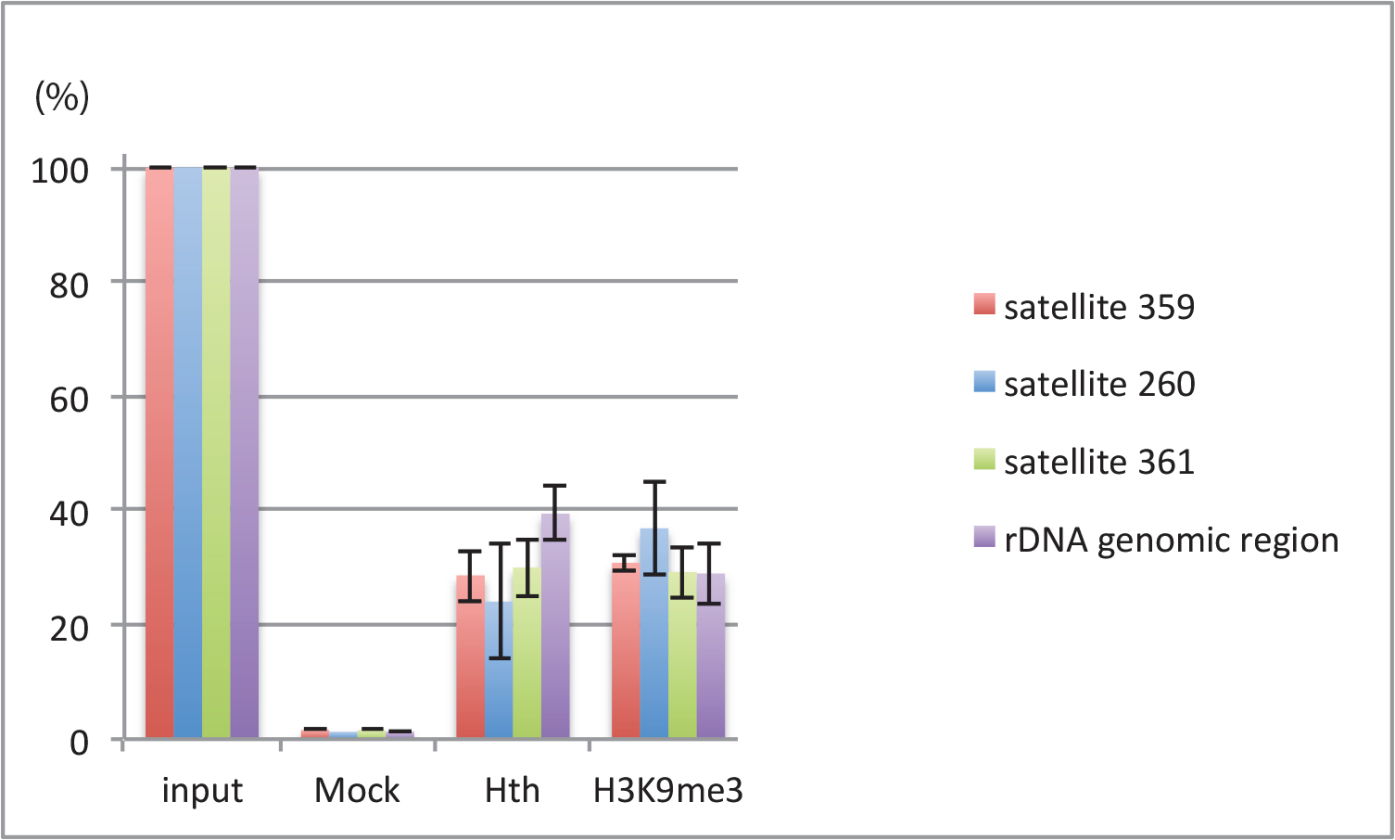
Homothorax binds to heterochromatic regions enriched in H3K9me3. ChIP experiments done with wild type embryos using anti-Hth and anti-H3K9me3 antibodies. The results show an enrichment of the 1.688 satellite region subfamilies as well as of the rDNA when the chromatin is immunoprecipiated with either antibody. Experiments were performed three times with three different DNA extractions.

In most of the eukaryotes, rDNA is tandemly repeated and embedded within heterochromatin. Previous reports showed that the rDNA genomic region in *Drosophila* is enriched in the H3K9me2 histone mark [34], and that this region is altered in Su(var)3–9 mutants, in which the levels of H3K9me2 are very low. We first decided to check if the rDNA genomic region is also enriched in H3K9me3. To this aim we performed chromatin immunoprecipitation experiments (ChIP) using a specific antibody for the H3K9me3 methyl mark. The result of such experiment is summarized in Fig. 4 (purple colour), and clearly demonstrates the presence of the H3K9me3 mark in the rDNA genomic region.

Because *hth* mutants show a general reduction in H3K9me3, we wonder if Hth could associate with the rDNA genomic region and somehow influence its tri-methylation in the Lys9 of H3. The result of the ChIP experiment using the specific anti-Hth antibody shows an enrichment of the rDNA region in the genomic fraction immunoprecipitated with Hth (see Fig. 4, purple colour) indicating that Hth associates with the rDNA region *in vivo*.

### *hth* mutant nuclei have multiple nucleoli

**Fig 5:**
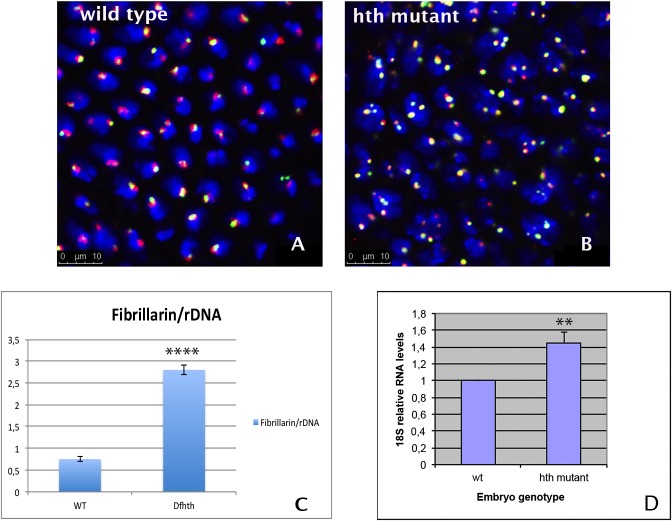
The nuclei of *hth* mutants have dispersed rDNA foci and ectopic nucleoli and produce more rRNA. A) Wild type nuclei stained with anti-Fibrillarin (red) and a specific probe that recognizes the rDNA genomic region (green). B) *hth* mutant nuclei stained as in A. The mutant nuclei exhibit an increased number of nucleoli with different sizes. C) Quantification of nuclear dots marked with both the rDNA probe and anti-Fibrillarin in wild type and mutant nuclei. 45 nuclei were quantified for each genotype. (p-value: 0,00010132, calculated using T-Test). D) qRT-PCR comparing the levels of 18S RNA transcripts in wild type and *Dfhth* mutant embryos. The experiment was performed three independent times to be significant (p-value: 0,01046, calculated using T-Test). Error bars in the graph show the standard deviation and have being calculated as in Fig. 1E. The experiment was normalized using the *bicoid (bcd)* mRNA.

We next checked if the observed binding of Hth to the rDNA is functional. To this aim we analysed by indirect immunofluorescence microscopy the distribution of the rDNA region in wild type and *hth* mutant nuclei using fluorescent in situ hybridization (FISH) with a specific DNA probe (see Material and Methods). Wild type nuclei displayed 1–2 rDNA sites in interphase (see Fig. 5 A, C), whereas in *hth* mutants the nuclei contained multiple dispersed rDNA foci (Fig. 5 B, C). We further analysed if these sites of miss-localized rDNA form ectopic nucleoli. Nucleolus only forms at NORs (nucleolus organizer regions) if the rRNA genes are active [37]. We stained the embryos with an antibody raised against Fibrillarin, a component of the rRNA processing machinery present in the nucleolus [38]. Wild type embryos displayed one or two nucleoli marked with both the rDNA probe and the anti-Fibrillarin antibody (Fig. 5 A, C). In contrast, Df*hth* mutant embryos contained multiple ectopic nucleoli mainly associated with the dispersed rDNA foci (Fig. 5 B, C). The size of the ectopic nucleoli is variable, and they display aberrant shapes.

The same result was obtained when *hth* ^*P2*^ mutant embryos were analysed (S5 Fig.). This result shows that in *hth* mutant embryos ectopic nucleoli form around dispersed rDNA clusters.

### *hth* mutants show higher rate of rRNA transcription

One possible outcome of the ectopic rDNA/Fibrillarin foci observed in the mutants is a deregulation in the synthesis of the rRNA. Fibrillarin is associated with RNAPolI and is an indicator of rRNA production [39]. The many foci marked with Fibrillarin in *hth* mutants might suggest a higher production of rRNA in the nuclei. To analyse this possibility we performed qRT-PCR in wild type and Df*hth* mutant embryos using specific primers for the 18S rRNA (see Material and Methods). As illustrated in Fig. 5 D, the levels of the 18S RNA are higher in *hth* mutants than in their wild type siblings. This result suggests that the many rDNA sites observed in *hth* mutants associated with Fibrillarin are active sites for transcription of rRNA.

### Satellite sequences display multiple Hth binding sites

In most eukaryotes, satellite and repetitive genomic sequences are embedded in heterochromatin. In *Drosophila*, each centromeric region contains different sets of satellite DNA sequences [40–44]. The X-chromosome contains a large array of the 1.688 satellite DNA (359-bp repeat unit) which is located in the centromeric region and in the nearby located pericentromeric heterochromatin. Autosomes also contain arrays of distinct subfamilies of the 1.688 satellite DNA in the pericentromeric heterochromatin. The 260-bp arrays are located at 2L and 353-bp arrays correspond to the 3L heterochromatin. We decided to first check if these satellite DNA regions of heterochromatic nature were enriched in H3K9me3 in wild type embryos. To this aim we performed ChIP assays with the specific anti-H3K9me3 antibody. The results are summarized in Fig. 4, and show a strong enrichment of all the tested 1.688 satellite arrays when the chromatin is precipitated with anti-H3K9me3.

**Fig 6:**
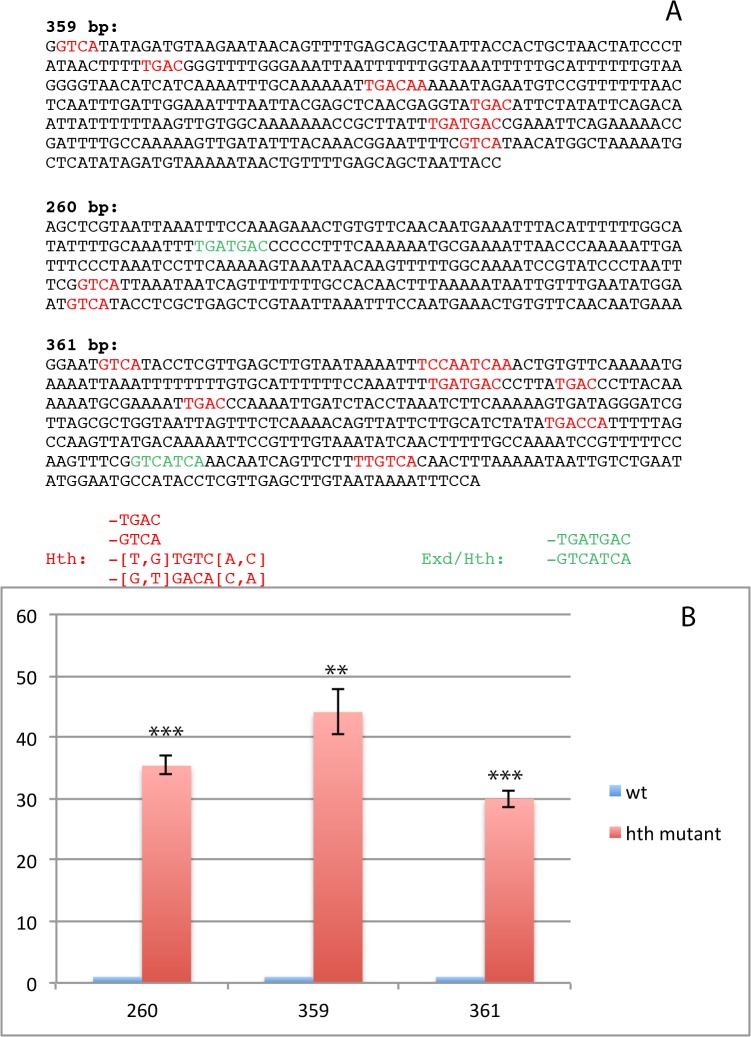
Homothorax binds to the distinct heterochromatic 1.688 satellite subfamilies. A) In silico binding sites for Hth alone or together with its partner Exd in the 3 satellite subfamilies. B) Result of the qRT-PCRs done with wild type and *hth* mutant embryos analysing the amount of RNAs transcribed from every satellite subfamily. The transcription is very much reduced in the mutant background (p-values are: 0,00064392 for 260 bp, 0,0020614 for 359 bp and 0,00073939 for 361 bp. All of them were calculated using the T-Test). Error bars show the standard deviation of each set of experiments that were calculated as in Fig. 1E. Every qRT-PCR was performed 3 times with RNA extracted from 3 different embryo collections.

We have previously shown that Hth binds to the 1.688 satellite subfamily flanking the centromeric and pericentromeric region of the X chromosome to facilitate its transcription [5]. To investigate if Hth is also able to bind to other 1.688 satellite subfamilies in autosomes we first searched in silico the distinct 1.688 satellite region subfamilies for binding sites for Hth, or Hth and its partner Extradenticle (Exd) [10, 45]. The result is shown in Fig. 6 A. Any of the searched subfamilies contain predicted binding sites for the analysed transcription factors. For the shortest 260 bp region the number of putative binding sites is 3, whereas 359 has 7 and 361 up to 12.

We then decided to perform ChIP experiments to demonstrate that Hth binds to these regions in vivo. The result is shown in Fig. 4. We find significant enrichment of all of the three satellite subfamilies in the chromatin immunoprecipitated with the anti-Hth antibody. This result suggests that Hth is able to bind to the three satellite subfamilies of 1.688 in the *Drosophila* embryos.

### Transcription of satellite repeats depends on the presence of Hth

The 1.688 satellite family has been shown to be heterochromatic [41, 46] and by ChIP experiments we have confirmed the presence of the H3K9me3 methyl mark in all the tested subfamilies (see Fig. 4).

It has been reported that the 1.688 satellite sequences are transcribed in *Drosophila* ovaries [47]. In an earlier work we were able to show that transcription of the 359-bp subfamily is dependent upon Hth [5]. To test if the presence of Hth is also necessary for the transcription of the other 1.688 subfamilies, we performed qRT-PCR and checked for the abundance of transcripts arising from the satellite subfamilies in wild type embryos and in embryos derived from mothers lacking the maternal component of *hth*. The result is summarized in Fig. 6 B. As shown there, the transcripts produced from all three subfamilies are reduced in abundance in the mutant background with respect to wild type embryos, indicating that Hth is necessary for the proper transcription of all the tested satellite RNAs.

### In the absence of Hth many heterochromatin regions acquire a more open conformation

In all of the heterochromatic genomic regions analysed in this work, we observe binding of Hth and enrichment in the H3K9me3 methyl mark, and at least for the 1.688 satellite family we were to show reduction of transcription in *hth* mutants. We wonder if the absence of Hth, which leads to a general reduction of H3K9me3 in the embryo, has an effect in the epigenetic state of these heterochromatic genomic regions. We favour this hypothesis because transcription of repetitive DNA elements has been shown in S. Pombe to be a pre-requisite for transcriptional silencing [48–50] and the epigenetic state of rDNA, and centromeres has been demonstrated to depend on non-coding RNAs [51–54]. Therefore, the observed reduction in satellite DNA transcription could in principle lead to a change in the epigenetic state of the analysed satellite regions.

We analysed this hypothesis by performing DNAseI sensitivity assays that help us to biochemically analyse the chromatin state of the genomic regions of interest [29]. This type of analysis distinguishes between open and close chromatin domains, in which the first are sensible to a DNAseI digestion, whereas the second are not.

Quantitative PCR was performed on native chromatin samples or on samples digested with DNAseI (50U) and the cycle threshold (Ct value) was calculated in function of the amount of DNA at the outset (unless otherwise stated, the ΔCt values mentioned refer to Ct [50U]-Ct [0U]).

For constitutively active genes that were used as positive controls (the “open” chromatin state), such as the *act5c* transcribed region, the ΔCt value was between 3 and 6 in wild type embryonic extracts. By contrast, heterochromatic regions were refractory to DNAseI treatment with a ΔCt value close to zero. If a given chromatin region changes its epigenetic state in the mutant background, this will result in a different degree of DNAseI digestion in the wild type versus the mutant condition.

**Fig 7:**
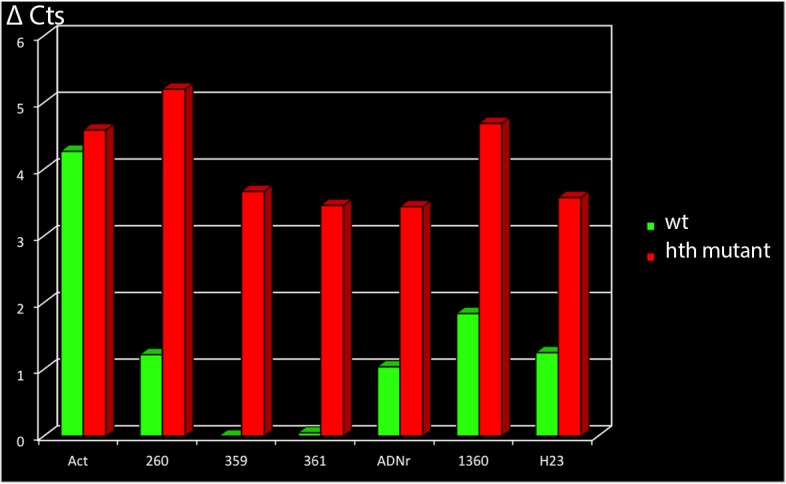
DNAseI sensitivity assay. The analysed genomic regions were subjected to a digestion with 50U of DNAseI. Open chromatin is accessible to the DNAseI and gets digested, whereas closed chromatin does not permit access to the enzyme and is refractory to the digestion. The actin region is an euchromatic one and has been used as a control. Every closed region analysed (including the H23 and 1.360 genomic regions) shows a more open chromatin state in the absence of Hth. The ordinate of the histogram represents ΔC_t_ = C_t_ (50U)- C_t_ (0U), being Ct (50U) the cycle threshold of the qPCR after treatment with 50U DNAseI and Ct (0U) the cycle threshold of the qPCR after treatment with OU DNAseI.

The results of the DNAseI sensitivity assays are summarized in Fig. 7. In wild type embryos, all the regions analysed showed a closed chromatin state, reflected by the fact that they are almost insensitive to a treatment with 50U DNAseI, in contrast to what happens with the control euchromatic actin region (see Fig. 7). This is in agreement with their heterochromatic condition. However, in *hth* mutant embryos, the same regions show a more open chromatin state and become sensitive to a digestion with 50U of DNAseI as shown in Fig. 7. This result is not only observed with the rDNA genomic region and all of the 1.688 satellite subfamilies, but also with other known heterochromatic regions analysed, as the 1.360 transposable element on chromosome four or the heterochromatic region H23 (22,000–24,000 located in the pericentromeric heterochromatin of chr. 2) [29].

These results indicate that the genomic regions that show enrichment of the H3K9me3 histone mark and binding of Hth acquire an open chromatin conformation in the absence of the transcription factor. Other heterochromatin regions in the embryonic genome show the same effect, although this phenomenon needs to be further analysed.

## Discussion

In the present work, we have extended or study on the epigenetic role of the transcription factor *hth* in *Drosophila*. Previously, we were able to show that *hth* mutants show defects in pericentric heterochromatin assembly that leads to aberrant early mitotic cycles. Here, we show that these mutant embryos display an overall reduction of the H3K9me3 histone mark throughout embryonic development.

The severe reduction in H3K9me3 observed in the mutant embryos seems not to be a consequence of a loss of the histone methyl-transferase Su(var)3–9 responsible for methylation of the histone H3 tail in the residue Lys9. In the first place because the same methyl-transferase is needed for the di-methylation of H3 in Lys9 which occurs in the mutant embryos. Secondly because a significant pool of the methyl-transferase used in the early embryonic stages is of maternal origin. The mother provides enough of the enzyme to fulfil the first methyl transfers until embryonic cycle 13–14. Our results rather suggest that the sole presence of the Su(var)3–9 methyl-transferase is not sufficient for the tri-methylation of the Lys9 of H3 to occur properly. The activity of Hth is also necessary in this process suggesting that transcription is an important step in the establishment of a subset of heterochromatin domains.

In the early *Drosophila* embryo H3K9 tri-methylation occurs after the di-methylation of the same residue [33]. We detect higher accumulation of the H3K9me2 mark in the mutants that show reduced levels of tri-methylation in the same residue. However, this accumulation does not occur in the chromatin, but in the cytoplasm, and corresponds to a form of H3K9me2 with a high molecular weight (150 kDa aprox.). Loyola and Almouzni suggested that there is a fraction of non-nucleosomal H3K9me2 present in the cell that is not susceptible of being further methylated by the Suv39 HMTase [55]. Our results in *Drosophila* embryos show that in *hth* mutants the cytoplasmic pool of H3K9me2 increases while the nucleosomal H3K9me3 decreases, suggesting that the cytoplasmic pool of H3K9me2 could be a source for H3K9me3 that depends on Hth for its incorporation into chromatin. If Hth per se is responsible for this incorporation, or this is a secondary effect of the lack of transcription described in the mutants needs to be further investigated.

We have been able to show that *hth* mutants show structural defects in all the subfamilies of the 1.688 satellite region tested. All of these DNA domains correspond to constitutive heterochromatin in *Drosophila*. It has been recently shown that transcription of non-coding RNAs of pericentric origin is necessary for heterochromatin reorganization during mouse development [56]. Binding of transcription factors to satellite sequences in mouse cells is also necessary for heterochromatin formation [6]. We have shown that Hth binds to three of the subfamilies of the 1.688 satellite sequence, and that loss of Hth reduces transcription of these and changes their chromatin conformation to a more open one. This result suggests a link between transcription of non-coding RNAs mediated by Hth and chromatin compaction, probably through incorporation of H3K9me3. There is, however, a small proportion of H3K9me3 in the chromatin whose incorporation does not depend on Hth and occurs even in the complete absence of the transcription factor. One possible explanation for this phenomenon is that other transcription factors could perform a similar function and compensate in part for the lack of Hth.

Other heterochromatic regions, like H23 and the 1.360 transposable element also seem to depend on Hth to acquire their heterochromatic conformation. We do not know yet how Hth influences the heterochromatin assembly of these regions, although we tend to think that its “opening” in *hth* mutants is directly associated with a reduction in the incorporation of H3K9me3.

In our work we also observe severe defects in the ribosomal DNA, a genomic region known to be embedded in heterochromatin in *Drosophila*. *hth* mutants show dispersed nucleoli and ectopic sites of rDNA. A similar phenotype has been previously described in mutants for the Su(var)3–9 methyl-transferase which show reduced levels of H3K9me2 [34]. We do not observe a reduction of H3K9me2 in our mutants, suggesting that the presence of H3K9me2 is not sufficient for the rDNA to assembly correctly. The rDNA is an intriguing genomic region. It has the most active site of cellular transcription and heterochromatic repressive structures. The establishment of the repressed rDNA has been linked to the assembly of the centric and pericentric heterochromatin of the cell. In fact, in cultured cells both genomic compartments, the perinucleolar heterochromatin and the major and minor satellite repeats are in close contact, and it has being reported that mutations that somehow affect the correct assembly of the heterochromatic rDNA also decrease heterochromatic marks (H3K9me3) at major and minor satellite repeats [57]. This physical proximity is also observed along the chromosomes in *Drosophila*, being the rDNA regions located close to pericentromeric repeats. Work done in *Drosophila* demonstrated that rDNA deletions result in reduced heterochromatin-induced gene silencing along the genome [58], again pointing to a close relationship between heterochromatic rDNA and other heterochromatin structures in the nucleus. Our results point to the same direction. We hypothesize that the general heterochromatin structures of the nucleus are the ones that influence the epigenetic state of rDNA.

Although formation of the close heterochromatic rDNA depends on transcription [59] this transcription is executed by the RNAPolI. So far, there is no report indicating that Hth interacts with the RNAPolI to facilitate its function. The fact that *hth* mutants show higher levels of 18S rRNA transcripts, probably arising from the fraction of rRNA genes that are now “open” for transcription, also points to an indirect role of Hth in rDNA condensation. Therefore, if Hth is not directly influencing RNAPolI transcription, the defects in the rDNA region that we observe in the mutants are most probably due to the effect that the neighbouring satellite sequences have on it. It is possible that diminution of the H3K9me3 histone mark, leading to loss of HP1, impedes correct spreading of heterochromatin to neighbouring genomic regions.

However, so far we cannot rule out the possibility that any of the many Hth isoforms present during embryonic development [60] is able to interact with PARP1 or other unknown protein that functions in the assembly of the heterochromatin in the rDNA region.

We have identified the transcription factor Hth as one of the key factors for transcription-dependent heterochromatin formation throughout embryonic development in *Drosophila*. A function of transcription factors in heterochromatin formation was first described by our group in *Drosophila* [5, 7], and later confirmed in mouse culture cells [6]. Hth mutants show overall reduced levels of H3K9me3 and de-condensation of all the constitutive heterochromatin structures analysed. In the mutants, the levels of chromatin-associated H3K9me2 are normal, but we detect reduced levels of non-coding RNAs of all the satellite sequences analysed.

Overall our data suggest that the di-methylation of H3 in K9 is not sufficient for many analysed heterochromatin genomic regions to condense properly. We propose that constitutive heterochromatin needs a higher degree of condensation that is achieved through transcription. The way this occurs in higher organisms needs to be further investigated.

## Supporting Information

S1 Fig Distribution of the H3K9me2 mark in *hth* ^*P2*^ mutant embryos. A, B) High levels of H3K9me2 are observed in the blastoderm stage of mutant embryos (green in A, white in B). C,D) A similar high accumulation of the H3K9me2 methyl mark are observed in germ band extended mutant embryos (green in C, white in D).
(TIF)

S2 Fig Western blot analysis of wild type embryos using anti- H3K9me2 antibody. Protein extraction was done to separate the cytosolic and chromatic fractions of the embryos. The chromatin extract displays an expected band of 17kDa. In the cytosol, a single band can be detected of approx. 150kDa.
(TIF)

S3 Fig Distribution of the H3K9me3 mark in *hth* ^*P2*^ mutant embryos. A,B) Mutant blastoderm embryo stained with an anti-H3K9me3 antibody. There is only a very faint distribution of the methyl marl in the topro3 dense region of the nuclei (green in A, white in B). C,D) The levels of H3K9me3 stay very low throughout embryonic development in *hth* ^*P2*^ mutant embryos. Only few cells show high accumulation of H3K9me3 in the topro3 dense region of the nucleus (green in C, white in D).
(TIF)

S4 Fig Distribution of the His2AvP mark in *hth* ^*P2*^ mutant embryos. A,B) Nuclei of mutant blastoderm embryos diplay high frequency of DNA breaks marked with the anti-His2AvP antibody (green in A, grey in B, see arrows).
(TIF)

S5 Fig Ectopic nucleoli in *hth* ^*P2*^ mutant embryos identified by the presence of the Fibrillarin protein. A-D) Different mutant *hth* ^*P2*^ embryos stained with an anti-Fibrillarin antibody. The number of spots per nucleus is always higher than in wild type embryos (compare with Fig. 5A) suggesting that the mutant nuclei have more loci of active rRNA transcription.
(TIF)
