# Supplementary figures and images for: The TALE Transcription Factor Homothorax Functions to Assemble Heterochromatin during Drosophila Embryogenesis
Source: PLoS One. 2015 Mar 20;10(3):e0120662. doi: 10.1371/journal.pone.0120662 (PMC4368669; doi:10.1371/journal.pone.0120662)

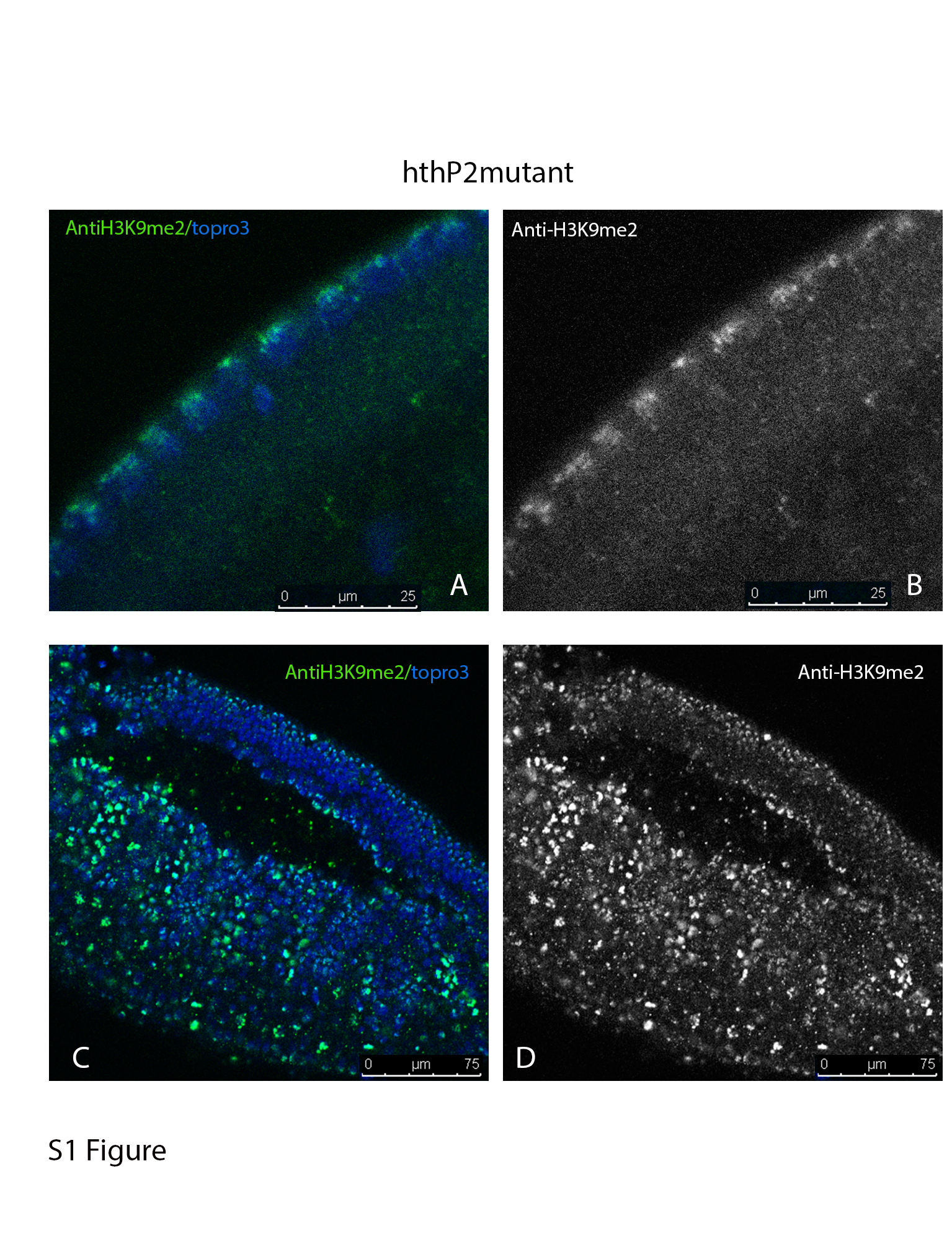

Supplement: S1 Fig — A, B) High levels of H3K9me2 are observed in the blastoderm stage of mutant embryos (green in A, white in B). C,D) A similar high accumulation of the H3K9me2 methyl mark are observed in germ band extended mutant embryos (green in C, white in D). (TIF) [file pone.0120662.s001.tif]

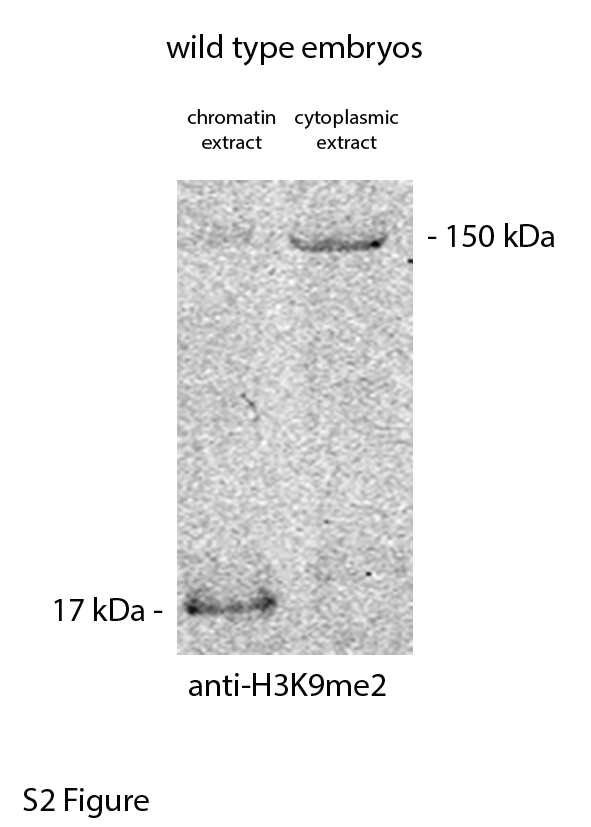

Supplement: S2 Fig — Protein extraction was done to separate the cytosolic and chromatic fractions of the embryos. The chromatin extract displays an expected band of 17kDa. In the cytosol, a single band can be detected of approx. 150kDa. (TIF) [file pone.0120662.s002.tif]

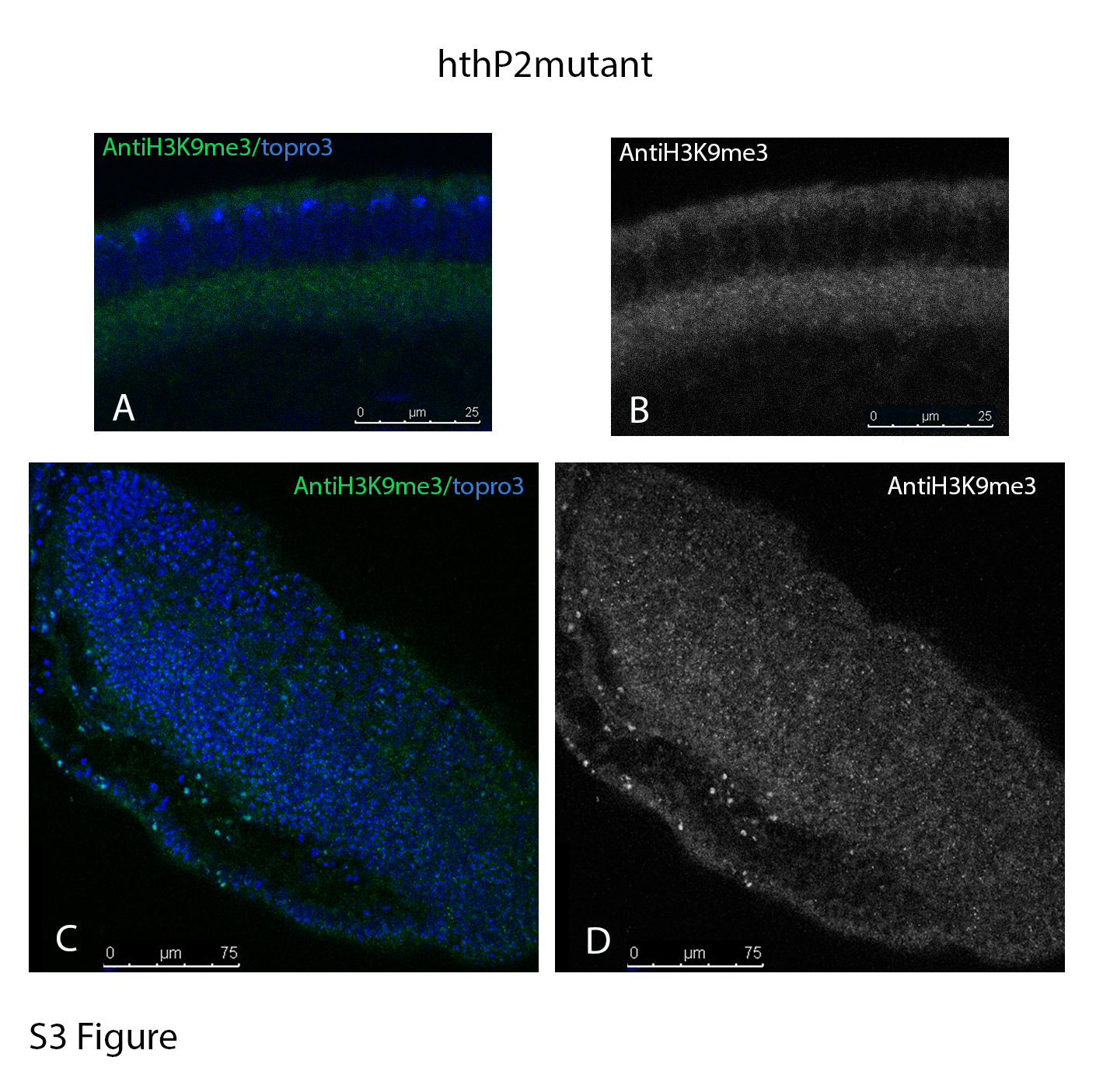

Supplement: S3 Fig — A,B) Mutant blastoderm embryo stained with an anti-H3K9me3 antibody. There is only a very faint distribution of the methyl marl in the topro3 dense region of the nuclei (green in A, white in B). C,D) The levels of H3K9me3 stay very low throughout embryonic development in hth P2 mutant embryos. Only few cells show high accumulation of H3K9me3 in the topro3 dense region of the nucleus (green in C, white in D). (TIF) [file pone.0120662.s003.tif]

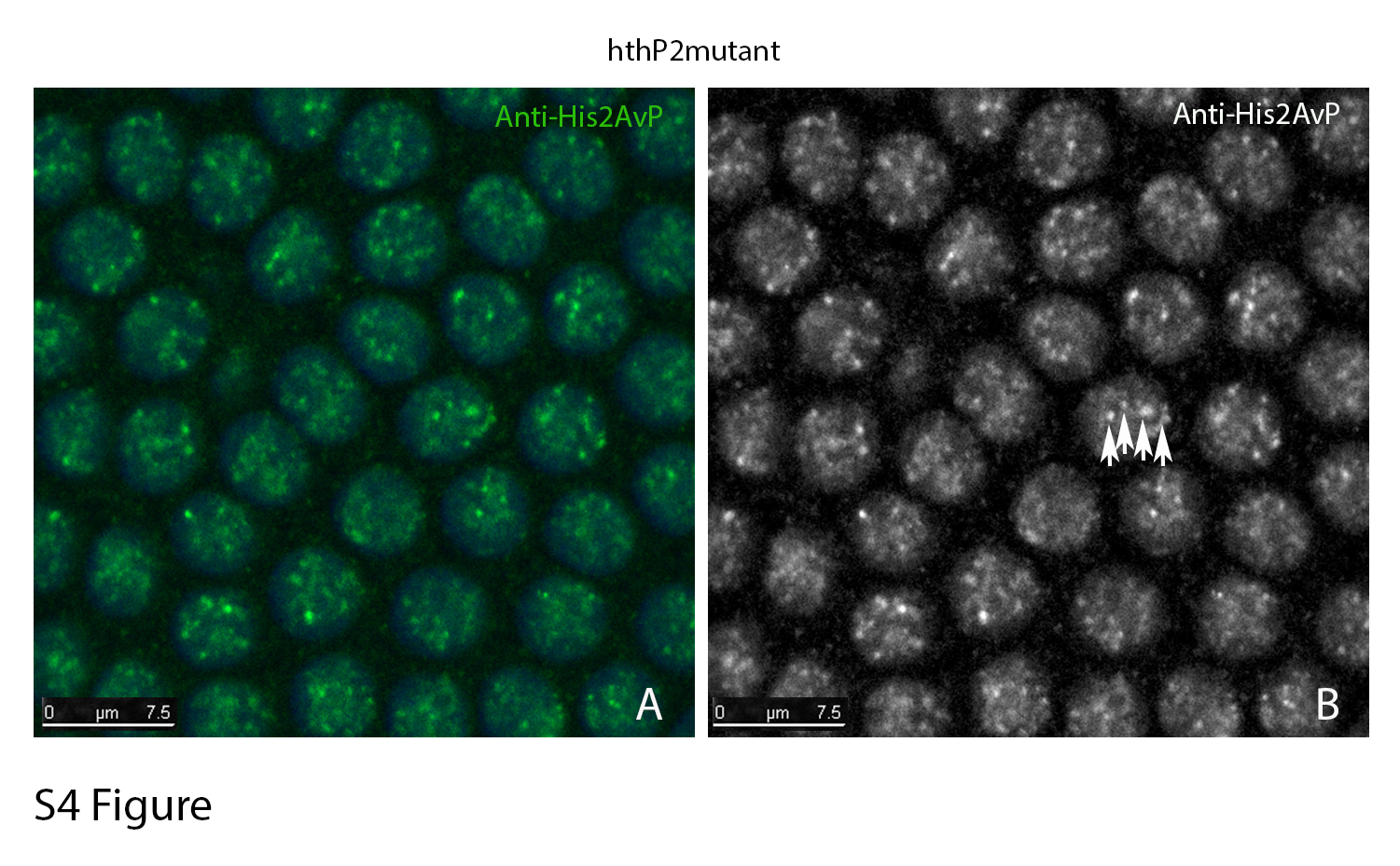

Supplement: S4 Fig — A,B) Nuclei of mutant blastoderm embryos diplay high frequency of DNA breaks marked with the anti-His2AvP antibody (green in A, grey in B, see arrows). (TIF) [file pone.0120662.s004.tif]

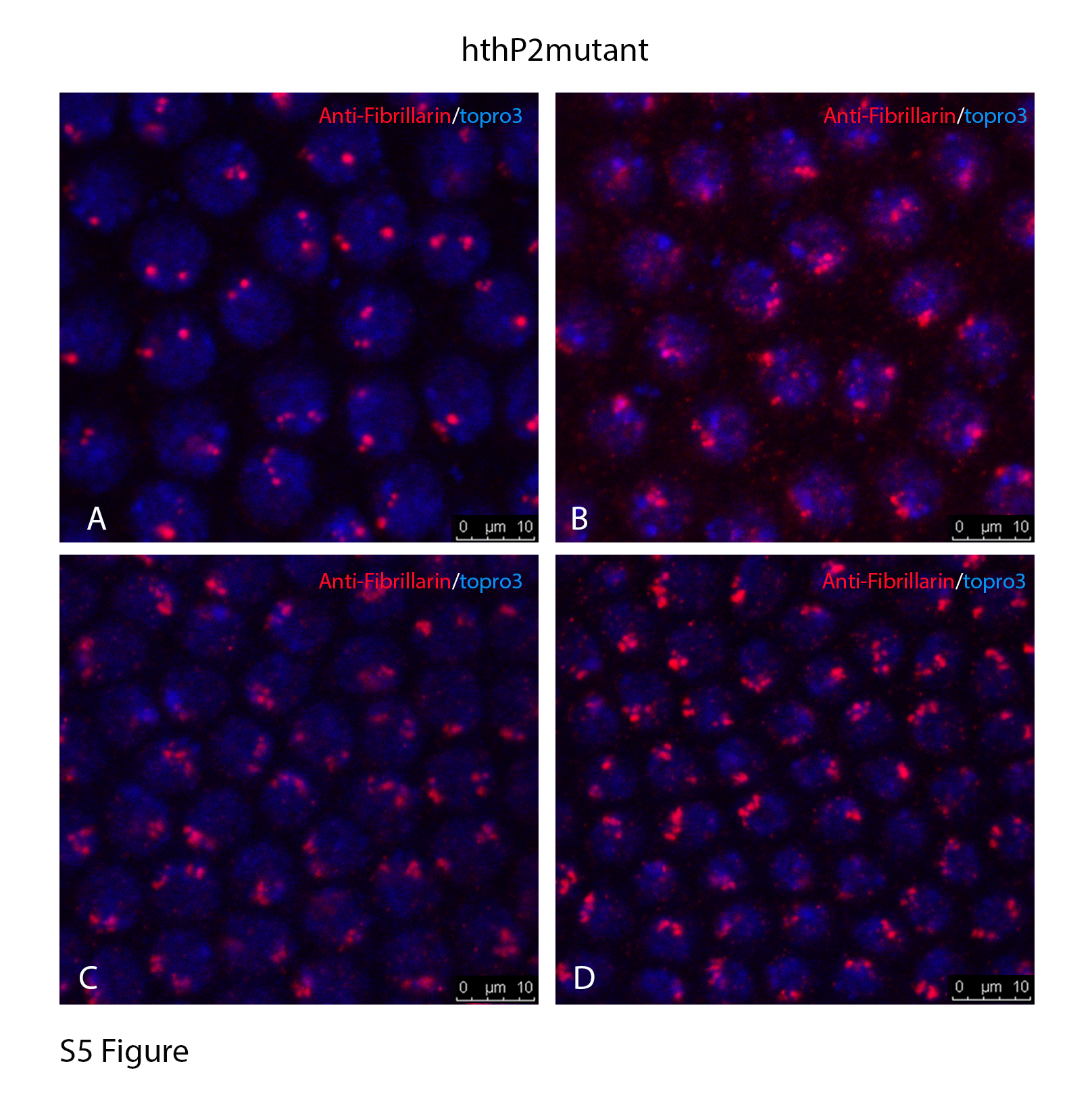

Supplement: S5 Fig — A-D) Different mutant hth P2 embryos stained with an anti-Fibrillarin antibody. The number of spots per nucleus is always higher than in wild type embryos (compare with Fig. 5A) suggesting that the mutant nuclei have more loci of active rRNA transcription. (TIF) [file pone.0120662.s005.tif]
